# New clinical score to diagnose nonalcoholic steatohepatitis in obese patients

**DOI:** 10.1186/1758-5996-3-3

**Published:** 2011-02-23

**Authors:** Fernanda BU Pulzi, Raul Cisternas, Murilo R Melo, Cristiane MF Ribeiro, Carlos A Malheiros, João E Salles

**Affiliations:** 1 Department of Internal Medicine, Endocrinology Unit of Irmandade da Santa Casa de Misericórdia de São Paulo - São Paulo, Postal Code 01221-020, Brazil; 2 Department of Surgery of Irmandade da Santa Casa de Misericórdia de São Paulo - São Paulo, Postal Code 01221-020, Brazil; 3 Department of Pathology of Irmandade da Santa Casa de Misericórdia de São Paulo São Paulo, Postal Code 01221-020, Brazil; 4 Department of Physiology of Santa Casa Medical School, São Paulo, Postal Code 01221-020, Brazil

## Abstract

**Background:**

Nonalcoholic fatty liver disease (NAFLD) is the most frequent disease associated with abnormal liver tests that is characterized by a wide spectrum of liver damage, ranging from simple macro vesicular steatosis to steatohepatitis (NASH), cirrhosis or liver carcinoma. Liver biopsy is the most precise test to differentiate NASH from other stages of NAFLD, but it is an invasive and expensive method. This study aimed to create a clinical laboratory score capable of identify individual with NASH in severely obese patients submitted to bariatric surgery.

**Methods:**

The medical records from 66 patients submitted to gastroplasty were reviewed. Their chemistry profile, abdominal ultrasound (US) and liver biopsy done during the surgical procedure were analyzed. Patients were classified into 2 groups according to liver biopsy: Non-NASH group - those patients without NAFLD or with grade I, II or III steatosis; and NASH group - those with steatohepatitis or fibrosis. The t-test was used to compare each variable with normal distribution between NASH and Non-NASH groups. When comparing proportions of categorical variables, we used chi-square or z-test, where appropriate. A p-value < 0.05 was considered statistically significant.

**Results:**

83% of patients with obesity grades II or III showed NAFLD, and the majority was asymptomatic. Total Cholesterol (TC)≥200 mg/dL, alanine aminotransferase (ALT) ≥30, AST/ALT ratio (AAR)≤ 1, gammaglutaril-transferase (γGT)≥30 U/L and abdominal US, compatible with steatosis, showed association with NASH group. We proposed 2 scores: Complete score (TC, ALT, AAR, γGT and US) and the simplified score, where US was not included. The combination of biochemical and imaging results improved accuracy to 84.4% the recognition of NASH (sensitivity 70%, specificity 88.6%, NPV 91.2%, PPV 63. 6%).

**Conclusion:**

Alterations in TC, ALT, AAR, γGT and US are related to the most risk for NASH. The combination of biochemical and imaging results improved accuracy to 84.4% the recognition of NASH. Additionally, negative final scores exclude the presence of an advanced illness. Using this score, the severity of fatty liver infiltration would be predicted without the risks associated with hepatic biopsy.

## Background

With the increased incidence of obesity worldwide, nonalcoholic fatty liver disease (NAFLD) has become a growing problem. NAFLD is a common and emergent condition now recognized as the most frequent cause of abnormal liver tests, especially in obese individuals [1,2]. It is characterized by a wide spectrum of liver damage, ranging from simple macro vesicular steatosis to steatohepatitis (NASH), cirrhosis or liver carcinoma [2-5]. In the general population, the estimated prevalence ranges from 3% to 24%, with most estimates in the 6% to 14% range. NAFLD is extremely common among patients undergoing bariatric surgery, ranging from 84% to 96%, these patients, 25% to 55% have NASH, 34% to 47% have fibrosis, and 2% to 12% have bridging fibrosis or cirrhosis [4].

Since the majority of NAFLD patients are asymptomatic, investigation usually begins after detection of abnormal liver enzymes on routine evaluation [4]. Serum aspartate aminotransferase (AST) and, more commonly, alanine aminotransferase (ALT) show mild to moderate elevation. The correlation with gammaglutaril-transferase (γGT) remains uncertain. Therefore, there is no overall correlation between the degree of liver enzyme elevation and the level of damage observed on histopathological analysis, beyond most of the patients with NAFLD show normal liver chemistries.

NAFLD appears to be most strongly associated with obesity and insulin resistance. There is some correlation between the severity of NAFLD and other features of metabolic syndrome, such as high triglycerides and low HDL, suggesting that NAFLD is an hepatic manifestation of metabolic syndrome [6-9].

Nonalcoholic steatohepatitis (NASH), which is the most severe histological form of NAFLD, is emerging as the most common clinically important form of liver disease in diabetes, obese patients and metabolic syndrome [6,10]. NASH has been associated with slight elevation of liver enzymes (mostly ALT and γ-GT) [10].

The "gold standard" for the diagnosis of NASH is liver biopsy, which allows us to differentiate simple bland steatosis from NASH [3,11]. However, liver biopsy is an expensive and invasive method associated with a low, but important procedure risk. Ultrasound (US), computerized tomography (CT), Magnetic resonance imaging (MRI), and H magnetic resonance spectroscopy (H MRS) are noninvasive methods and should be preferred. One limitation being that US does not provide reliable quantitative information [11]. Both the CT and MRI techniques are nonspecific and can be affected by processes such as excessive glycogen storage, edema and inflammation [11].

The aim of our study was to create a clinical laboratory score capable of identify individual with most risk for NASH in severely obese patients submitted to bariatric surgery. Using this score, the severity of fatty liver infiltration would be predicted without the risks associated with hepatic biopsy.

## Methods

We reviewed the medical records of all patients submitted to gastroplasty at the Hospital da Irmandade da Santa Casa de Misericordia de São Paulo, between March 2004 and April 2006 and who had intra-operative liver biopsies at the time of Roux-en-Y distal gastric bypass. The inclusion criteria for the study were: patients with BMI ≥ 35 Kg/m^2 ^that underwent the surgical treatment without any other adventitious causes of hepatopathy as alcoholism or viral hepatitis. The exclusion criteria were the ingestion of more than 20 g of alcohol per day or regular ingestion of drugs known to produce steatosis (glucocorticoids, tamoxifen, amiodarone) or any other condition with concomitant liver disease (viral hepatitis, autoimmune hepatitis, primary biliary cirrhosis, hemochromatosis or Wilson's disease). According to these criteria, 66 patients were selected for the study. The following data were evaluated: preoperative conditions, gender, age, body mass index (BMI), presence of hypertension and diabetes mellitus, chemistry profile including aspartate aminotransferase (AST), gammaglutaril-transferase (γGT), alkaline phosphatase (ALP), total cholesterol (TC), low density lipoprotein (LDL), high density lipoprotein (HDL), triglycerides, fasting glucose (FG) and abdominal US.

The diagnosis of metabolic syndrome followed the criteria established by the International Diabetes Federation (IDF) 2005 [12].

NAFLD was diagnosed by ultrasonography using an abdominal probe at 2-5 MHz. Longitudinal, sub costal, ascending, and oblique scans were performed. The ultrasonographic criteria that were used to diagnose fatty liver included liver and kidney echo discrepancy, presence of increase liver echogenicity (bright), echo penetration into the deep portion of the liver, and clarity of liver blood vessel structures.

The hepatic biopsy was performed intra-operatively and analyzed according to Brunt criteria [13], as is shown in Table 1. We classified our patients in two groups: Non-NASH group (patients without NAFLD or with grade I, II or III steatosis without hepatitis) and NASH group (patients with steatohepatitis or fibrosis). Demographic, laboratory and imaging data were compared between both groups.

**Table 1:** Classification into 2 groups according to liver biopsy: NASH and Non-NASH

*Brunt's score*	*n (%)*	*Study Score*
Absent NAFLD	11 (11.6)	Non-NASH Group
Steatosis I	21 (31.8)	
Steatosis II	11 (16.6)	
Steatosis III	10 (15.1)	
Fibrosis I	9 (13.6)	NASH Group
Fibrosis II	3 (4.5)	
Fibrosis III	0 (0)	
Fibrosis IV	1 (1.5)	
Total	66 (100)	

Patients without NAFLD or with steatosis, grade I, II or II were classified as Non-NASH group. Patients with NASH were classified as NASH group. The classification of NAFLD was according to Brunt's score.

The t-test was used to compare each variable with normal distribution between NASH and Non-NASH groups. When comparing proportions of categorical variables, we used chi-square or z-test, where appropriate. A p-value < 0.05 was considered statistically significant.

In order to propose a score system that is simple to use, we aimed to define cut-off levels for variables that would improve the model. We evaluated all continuous variables with p < 0.300 in the group comparisons using ROC (Receiver Operating Characteristics) curve analysis, to evaluate the sensitivity and specificity of cut-off levels. We included in our models all variables that presented the average of sensitivity and specificity of 60% or above.

If both AST/ALT ratio (AAR) and one of the enzymes that compose the index were to be included in the models, a kappa statistics (agreement) above 70% would indicate that only one would be necessary for the model.

These variables and cut-off levels were employed to classify patient's results and used to perform chi-square tests. We used 2 × 2 tables to calculate sensitivity, specificity, positive (PPV) and negative (NPV) predictive values. We used SPSS 17.0 (SPSS, USA) and MS-Excel 2003 (Microsoft, USA) to perform calculations.

## Results

We evaluated 66 individuals (11 males), with mean (SD) of 40.7(10.9) years of age and BMI of 46.4(6.5) kg/m^2^. Metabolic syndrome was present in 37 patients and type 2 DM in 10 subjects (7 women).

Males presented higher BMI (51.2 ± 5.3 kg/m^2^) than women (45.6 ± 6.3, p = 0.009, t-test), and similar age (males 38.8 y ± 9.3; women: 41.1 ± 11, p = 0.484, t-test). The proportion of cases with metabolic syndrome was also similar (males 7/11 cases; women 30/44 cases, p = 0.775, chi-square test).

Metabolic syndrome occurrence was similar in the NASH group (8/12 cases) when compared to Non-NASH group (29/43; p = 0.960, chi-square test). All patients presented abdominal waist values above the standards established. Age, BMI, sex, waist circumference, hypertension, lipid and hepatic enzymes (AST and ALT) measurements showed no association with NASH. However, age, TC, AST, ALT, the AST/ALT ratio (AAR) and γGT hap p values below 0.3 (Table 2) and a ROC curve analysis was performed to establish cut-off values (Table 3).

**Table 2:** Clinical, Laboratory and Imaging characteristics of the groups.

	*Non-NASH group* *AVG (± SD)*	*NASH group* *AVG (± SD)*	*P*
Gender: % women	84.9	76.9	0.503
Age ( years)	39.8 (± 10.4)	44.6 (± 12.5)	0.216*
BMI ( Kg/m^2^)	46.3 (± 6.45)	47.0 (± 6.72)	0.714
HAS (% positive)	58.5	46.2	0.423
DM (% positive)	13.2	23.1	0.374
CT (mg/dL)	195.4 (± 37.28)	210.2 (± 22.3)	0.094*
LDL (mg/dL)	120 (± 35)	125.8 (± 22)	0.488
TG (mg/dL)	141.3 (± 57.5)	163 (± 111)	0.544
HDL (mg/dL)	49.0 (± 11.9)	48.9 (± 7.9)	0.968
FG (mg/dL)	103.2 (± 23.9)	100.5 (± 11.6)	0.589
AST (IU/L)	23.6 (± 9.2)	31.6 (± 22.4)	0.248*
ALT (IU/L)	24.5 (± 13.1)	44.8 (± 45)	0.151*
GGT (IU/L)	38.2(24.4)	46.5(18.5)	0.229*
AAR	1.000	0.7677	0.047**
ALP (IU/L)	170.2 (± 79.7)	159.6 (± 56.5)	0.659
SM (% positive)	67.4	66.7	0.960
US (% abnormal)	47	77	0.048**

Data is expressed as Mean ± SD (except for AAR that has a non-parametric distribution).

**Table 3:** Cut-off and ROC curve analysis of each marker that was included in the score systems.

*Test*	*Cut-off*	*AUC (95% interval)*	*P*	*Sensitivity*	*Specificity*
TC	200 mg/dL	66.1% (51.2 - 81%)	0.091	83.3%	58.1%
AAR	1.000	68.8% (52 - 86%)	0.047	83.3%	46.7%
ALT	30 IU/L	70.1% (53 - 87%)	0.034	66.7%	71.1%
GGT	30 IU/L	65.5% (49 - 82%)	0.084	90.9%	44.2%
US	Abnormal	n/a	n/a	76.9%	46.9%

An abdominal US was available for 62 patients, presenting normal (or non-steatosis) more frequently in Non-NASH (26/49) than in NASH (3/13; p = 0.048, chi-square). Abdominal US presented sensitivity and specificity of 76.9% and 46.9%, respectively, to identify NASH.

**Figure 1:**
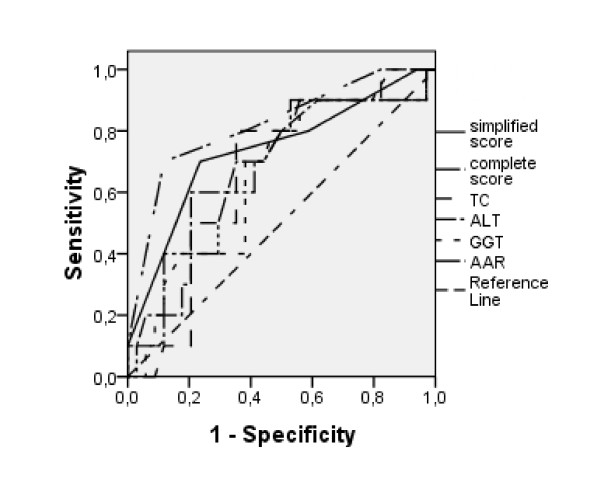
**ROC curves showing the sensitivity and specify to identify severe steatosis**.

Using ROC curve analysis, we established the following cut-off levels (equal or above being indicative of NASH): TC of 200 mg/dL, ALT of 30 IU/L and γGT of 30 IU/L (Figure 1). For AAR, a value equal of below 1 was indicative of NASH. Although the area under curve (AUC) of the ROC curve was not always significant (table 3), these tests presented sensitivities ranging from 67% to 83% with specificity of 44% to 71% (average of sensitivity and specificity ranged from 62% to 71%). The kappa statistics of AAR ≤ 1 and ALT ≥ 30 IU/L in defining NASH patients was 29.4% (p = 0.201), therefore both were included in the model.

Both age and AST were not included in the model for failing to present a cut-off level with average of sensitivity and specificity of 60% or greater. Age of 36.5 years provided the best discrimination with sensitivity of 69.2% and specificity of 43.4%. The best cut-off for AST was 17 IU/L with sensitivity of 91.7% and specificity of 22.2%.

Low accuracy prevents the use of each test alone. Therefore, we decided to evaluate two score systems (with and without US, as complete and simplified scores). In the complete score, we assigned 1 point for each of the following parameters: TC ≥200 mg/dL; AAR ≤ 1; ALT ≥30 IU/L; γGT ≥30 U/L and steatosis at abdominal US, with a total of 5 possible points. In the simplified score, US were not evaluated; therefore, a total of 4 points was possible.

**Figure 2:**
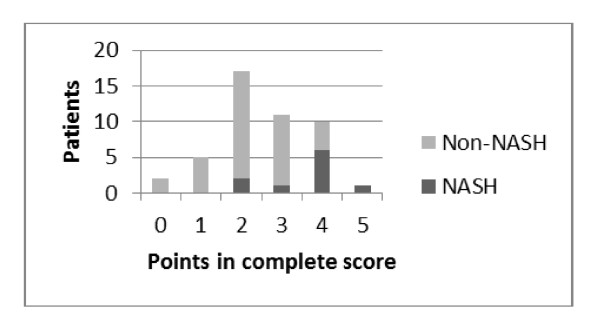
**Number of patients with NASH according to the number of points of a simplified score system**.

**Figure 3:**
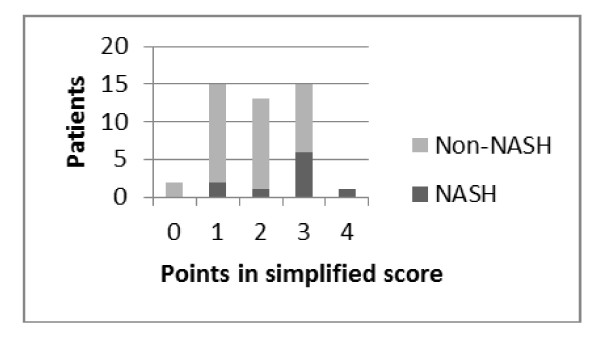
**Number of patients with NASH according to the number of points in complete score system**.

Both score systems were evaluated using ROC analysis (Figure 1). The complete score could be evaluated in 10 NASH patients and 35 Non-NASH patients (Figure 2) and presented an area under curve of 82.4% (CI 95%:67% - 97.8%, p = 0.002, ROC). The simplified score was evaluated in 10 NASH patients and 36 Non-NASH patients (Figure 3) and presented an area under curve of 73.1% (CI 95%= 54.1% - 92.0%, p = 0.027) to identify NASH. In table 4, we show sensitivity, specificity, positive and negative predictive values with different points in both scores. When no points are obtained, regardless of the score system employed, sensitivity and positive predictive value to identify NASH are equal to zero.

**Table 4:** Sensitivity, specificity, positive and negative predictive values and accuracy to identify NASH at liver biopsy.

	*≥3 out of 4 points (simplified score)* *(n = 52)*	*4 out of 4 points (simplified score)*	*≥ 4 out of 5 points (complete score)* *(n = 51)*	*5 out of 5 points (complete score)*
Sensitivity (%)	70	10	70	10
Specificity (%)	75	100	88.6	100
PPV (%)	43.8	100	63.6	100
NPV (%)	90	80	91.2	79.5
Accuracy (%)	73.9	80.4	84.4	80

## Discussion

Despite the high prevalence and severity of hepatic illness, NAFLD remains underdiagnosed. This is in part because of limitations in clinical diagnosis as a consequence of few symptoms, lack of accurate laboratory markers and restrictive indication of hepatic biopsy [1-3].

Thus, identifying noninvasive approaches for distinguishing simple hepatic steatosis from NASH in patients with NAFLD is critical given the increasing incidence and prevalence of this disease in our society.

Liver biopsy is the only way to confirm or exclude the diagnosis of NASH [3,11], giving valuable information regarding staging, prognosis and progress of this pathologic condition. However, serious complications have been communicated in percutaneous liver biopsy, they included retroperitoneal bleeding, and bile leak and, as many as one third of patients experiment strong pain [14,15]. The major contraindication to percutaneous liver biopsy is significant coagulopathy and relative contraindication to this procedure is obesity. Therefore, these kinds of liver biopsies not only incur increased risk to the patient, but also it is not possible to perform liver biopsies in all patients with NAFLD to exclude the severe form of this disease. Recent developments of laparoscopy bariatric surgery for obesity have increased the number of intraoperative liver biopsies.

Our study demonstrated that 83% of patients with obesity grades II or III showed NAFLD, and the majority was asymptomatic.

Some studies have shown that obesity is a metabolic illness closely associated with both steatosis and NASH, and the severity of the hepatic illness is related to the increase of BMI. In disagreement with this statement of Dixon et al [16], Beymer et al [17] showed that BMI did not differ between groups with and without NASH in morbidly obese patients, and that insulin resistance, hypertension, and elevated ALT were independently predicted the presence of NASH. In our study BMI did not differ between groups with and without NASH.

Serum liver enzyme abnormalities are primarily restricted to elevations of ALT. The majority of elevations are mild (< 5× the upper normal limit), and exist in all degrees of NAFLD. However, there isn't correlation between the degree of liver enzyme elevation and the level of damage observed on histopathological analysis, as has been seeing in most of the patients with NAFLD that present normal laboratory values, being not useful as a differential marker. Our study ALT was correlated to the severity of the hepatic damage.

In contrast to patients with alcohol-induced liver disease, the AST/ALT ratio (AAR) among NAFLD patients is usually less than 1 [3]. In our study, we confirmed this finding. Evidence that this ratio may reverse with advanced fibrosis or cirrhosis has been reported [3,11,15]. AAR greater than 1 can be an independent risk factor for advanced fibrosis among patients with biopsy-proven NASH according some studies [18,19].

The role of γGT, as a molecular marker for disease severity and diagnostics is still obscure in NAFLD. Nevertheless, Sakugawa's [20] data showed that was a significant difference between γGT level and the severity of liver fibrosis. Our results show that γGT ≥ 30 IU/L is an adequate marker of NASH.

Thus, considering the markers of liver damage, γGT, ALT and AAR were correlated to the severity of the hepatic damage. We believe that aminotransferases abnormalities probably occur at an earlier stage, different from γGT that would require a greater hepatic damage to be altered.

High fasting glucose levels and lipid alterations, such as hypertriglyceridemia and low HDL are laboratory markers of insulin resistance [21,22]. In our study, these markers presented no correlation with the severity of the liver disease.

The sensitivity and specificity of US is considered reasonable for detecting liver steatosis. However, the sensitivity of US might be lower in mild fat infiltration and is difficult in the morbidly obese patient because of excessive fat which might take us to an unreliable result [3]. Liang et al [23] disagree with the above, since they have shown that US use may be acceptable in the morbidly obese patients. We would argue that as US depends of the equipment available, the software used for analysis and the operator, satisfaction with the obtained results can vary between different centers. Our analysis did not consider the degree of hepatic alteration, but simply showed whether steatosis was present. US alone was able to detect 76.9% of the cases but presented a low specificity (46.9%), however, when associated with other parameters, such as the 5-points (complete) score, its sensitivity increased to 88.6% while maintaining reasonable sensitivity (70%) for the more serious cases. Therefore, in severely obese patients, in whom US specificity is lower, the inclusion of the score significantly improved the identification of these cases. Other scores have been described aiming at the correlation between isolated laboratory findings, in the attempt of improving the non-invasive diagnosis of NAFLD.

Palekar [19] proposed a diagnostic model using both clinical and laboratory data to enhance distinguishing patients with simple steatosis from those with NASH. Patients with three or more of the following characteristics are more likely to have NASH than simple steatosis: female gender, age ≥ 50 years, AST > 45 IU/L, BMI ≥ 30 Kg/m2, AAR ≥ 0.8 and hyaluronic acid ≥ 55 mcg/L. ROC curve analysis showed accuracy of 0.763. The presence of three or more factors showed sensitivity, specificity, PPV and NPV of 73.7%, 65.7%, 68.2% e 71.4%, respectively.

Sakugawa [20] showed that type VI collagen 7S domain ≥ 5.0 ng/mL and hyaluronic acid ≥ 43 ng/mL were markers of liver fibrosis that had a high positive predictive value ( 86% and 92%, respectively) and high negative predictive value ( 84% and 78%, respectively).

Angulo [18] identified independent predictors of liver fibrosis: age > 45 years, the presence of obesity or type 2 diabetes mellitus, and AST/ALT ratio > 1.

In this study we propose a diagnostic model using clinical, laboratory and imaging data to improve the differential diagnosis of patients who belong to the Non-NASH group from those are part of NASH group. Patients with three or more of the following characteristics are more likely to have NASH (sensitivity 70%, specificity 75%, NPV 90%, PPV 43.8%): TC ≥ 200 mg/dL, ALT ≥ 30 IU/L, γGT ≥ 30 IU/L and AAR ≤ 1. If we add the US, and the patient shows four or more of the five criteria, the sensitivity is 70%, specificity is 88.6%, NPV 91.2% and PPV 63.6%. This composite index seems to be a good discriminator to identify the NAFLD patients with more severe disease.

As can be seen here, the sensitivity, specificity, PPV and NPV obtained by the measurements we propose in this study are better than the ones previously reported.

All patients from NASH group presented at least one point in the simplified score and complete score, suggesting very low risk of an advanced hepatic illness in negative scores.

Identifying patients at risk may assist physicians in planning the diagnosis and treatment, selecting those patients who really need liver biopsy. Furthermore, additional stratification for advanced NAFLD may be helpful to identify those patients who are at risk for disease progression and could benefit from future medical therapy or enrollment in clinical trials.

The score system suggested by this study can be easily applied. It routine biochemical tests (TC, ALT, AAR and γGT) in its simplified version, or it can be accompanied by US in the complete version. It is highly sensitive for advanced hepatic illnesses and can provide the practitioner with information to offer a more adequate treatment.

The present study provided evidence of applying a score test to evaluated non alcoholic liver disease in obese patients. However, our study presents some limitations such as, the reduced number of patients. We also studied subjects with obesity grade III, and some of them are metabolic healthy, meaning that they don't have any component of metabolic syndrome. Future study introducing an independent test group in order to validate the proposed score test would improved the present study.

## Conclusion

In conclusion, alterations in TC, ALT, AAR, γGT and US are related to a higher risk for NASH. The combination of biochemical and imaging results improved accuracy to 84.4% the recognition of NASH (sensitivity 70%, specificity 88.6%, NPV 91.2%, PPV 63. 6%). Additionally, negative final scores exclude the presence of an advanced illness. Although not essential, US is simple, non-invasive and a low-cost method, which can improve the score system in the can diagnosis of NASH or in the recognition of co-morbidities. Using this score, the severity of fatty liver infiltration would be predicted without the risks associated with hepatic biopsy.

## Competing interests

The authors declare that they have no competing interests.

## Authors' contributions

FBUP, RC, JENS conceived and designed the study. FBUP, RC, JENS and MRM participated in data analysis and interpretation of results. CMFR contributed in pathological analysis and classification of NAFLD or NASH. CAM surgeon that led the surgical procedure. All the authors read and approve the final manuscript.
